# The Autophagy Marker LC3 Is Processed during the Sperm Capacitation and the Acrosome Reaction and Translocates to the Acrosome Where It Colocalizes with the Acrosomal Membranes in Horse Spermatozoa

**DOI:** 10.3390/ijms24020937

**Published:** 2023-01-04

**Authors:** Ines M. Aparicio, Patricia Rojo-Domínguez, Alba Castillejo-Rufo, Fernando J. Peña, Jose A. Tapia

**Affiliations:** 1 Department of Physiology, Institute of Molecular Pathology Biomarkers (BICOMCEL), University of Extremadura, 10003 Cáceres, Spain; 2 Laboratory of Spermatology, Veterinary Teaching Hospital, University of Extremadura, 10003 Cáceres, Spain

**Keywords:** autophagy, LC3, horse, spermatozoa, capacitation, acrosome reaction, A23187, STF-62247, chloroquine, E-64d, 3-methyladenine

## Abstract

Despite its importance in somatic cells and during spermatogenesis, little is known about the role that autophagy may play in ejaculated spermatozoa. Our aim was to investigate whether the molecular components of autophagy, such as microtubule-associated protein 1 light chain 3 (LC3), are activated in stallion spermatozoa during the capacitation and acrosome reaction and if this activation could modulate these biological processes. To analyze the autophagy turnover, LC3I and LC3II proteins were assessed by western blotting, and the ratio between both proteins (LC3II/LC3I) was calculated. In somatic cells, this ratio indicates that autophagy has been activated and similar LC3 processing has been described in mammalian spermatozoa. The subcellular localization of autophagy-related proteins was assessed by immunofluorescence with specific antibodies that recognized Atg16, Beclin-1, and LC3. The colocalization of acrosomal membranes (PNA) and LC3 was studied by confocal microcopy, and the acrosome reacted cells were quantified by flow cytometry. The incubation of stallion sperm in capacitating conditions (BWW; 3 h) significantly increased LC3 processing. This increment was three to four times higher after the induction of the acrosome reaction in these cells. LC3 was mainly expressed in the head in mature ejaculated sperm showing a clear redistribution from the post-acrosomal region to the acrosome upon the incubation of sperm in capacitating conditions (BWW, 3 h). After the induction of the acrosome reaction, LC3 colocalized with the acrosome or the apical plasmalemma membranes in the head of the stallion spermatozoa. The inhibition or activation of autophagy-related pathways in the presence of autophagy activators (STF-62247) or inhibitors (E-64d, chloroquine) significantly increased LC3 processing and increased the percent of acrosome reacted cells, whereas 3-methyladenine almost completely inhibited LC3 processing and the acrosome reaction. In conclusion, we found that sperm capacitation and acrosome reaction could be regulated by autophagy components in sperm cells ex vivo by processes that might be independent of the intraluminal pH of the acrosome and dependent of LC3 lipidation. It can be speculated that, in stallion sperm, a form of noncanonical autophagy utilizes some components of autophagy machinery to facilitate the acrosome reaction.

## 1. Introduction

Macroautophagy (hereafter autophagy) is an evolutionary highly conserved cellular process among eukaryotes. Autophagy is involved in the maintenance of intracellular homeostasis through the degradation of most long-lived proteins and entire organelles. When canonical autophagy is activated in yeast and mammalian cells, a membrane cisterna called phagophore expands and encloses a portion of cytoplasm, resulting in the formation of the autophagosome with a double bilayer membrane surrounding the cargo. In the following step, the outer membrane of the autophagosome fuses with the lysosome, resulting in a degradative structure, termed as autolysosome or autophagolysosome, where hydrolytic enzymes, supplied by the lysosome, degrade the cytoplasm-derived materials together with the inner membrane of the autophagosome [1,2]. Depending on the cellular component degraded, it results in the removal of damaged organelles, the generation of free amino acids and energy sources, the removal of intracellular pathogens, the capture and processing of self-antigens for endogenous antigen presentation specific context, and others [2,3,4].

Phagophore, autophagosome, and autophagolysosome formation is finely regulated by at least 30 autophagy-related proteins (Atg). Atg genes were originally described in yeast, and their orthologs have been isolated and functionally characterized in mammals [1,2,5]. Atg1 and Beclin-1 (mammalian ortholog of Atg 6) participate in the early stages of this process. Further, protein associations among Atg5, Atg12, Atg16, and the lipidation of Atg8 induce the autophagosome formation [2].

Microtubule-associated protein light chain 3 (LC3) is the mammalian equivalent of yeast Atg8 and exists in two forms, LC3I and LC3II, being the second processed form of the first. LC3I is a 16–18 kDa polypeptide normally found in cytosol, whereas LC3II (12–14 kDa), which is conjugated with phosphatidylethanolamine (PE) by the action of ATG3/4/7 proteins, is present on membranes and autophagosomes and much less in autolysosomes [6]. LC3II plays crucial roles in autophagy, such as the elongation and completion of the autophagosome and the transfer of cargo to these structures by interacting with other signaling proteins, including the complex p62/SQSTM1 (sequestosome 1) [1,2]. Although the molecular weight of LC3II is higher than that of LC3I due to the addition of PE, LC3II migrates faster than LC3I in SDS-PAGE, probably because of its extreme hydrophobicity [7]. LC3II has been widely used to study canonical autophagy in somatic cells and gametes, and it has been considered as an autophagosomal marker in mammals and is used to monitor autophagy progression [6,7].

LC3 can also experiment with alternative processing forms and different regulatory mechanisms during the non-canonical forms of apoptosis [8]. For example, in addition to the canonical C-terminal lipidation of LC3 with PE, in some situations, LC3 could be conjugated with phosphatidylserine (PS). This is typically associated with LC3-associated phagocytosis (LAP), where LC3 conjugation to phagosomes involves PS conjugation [9].

In animal and human reproduction, autophagy-related processes seem to exert important roles [10]. One of those is played during gametogenesis, in which canonical autophagy is involved in the regulation of cell maturation and cell renewal during spermatogenesis, acting as a partner of apoptosis and phagocytosis [11,12]. Autophagy markers, such as LC3II, Atg7, and autophagosomes, have been described in rat and mice spermatogenic cells [10,11,13,14] and increased considerably during critical steps of gamete maturation [15]. In some of these studies, autophagy, measured as the ratio of LC3II/LC3I, increased significantly after 12 h of culture under metabolic stress [13], after testes heat treatment [11], or after electromagnetic radiation [14], showing that autophagy is not only activated by physiological conditions in the spermatogenesis, but it is also playing a supporting role in gamete production under stressful situations.

Interestingly, a number of reports have shown a critical function for Atg7 and other related components in the process of acrosome biogenesis in different species during spermiogenesis [16,17,18], supporting the autolysosome origination hypothesis for acrosome formation. Autophagy is also required for spermatozoa flagella biogenesis and cytoplasm removal during spermiogenesis [10].

In post-fertilization events, autophagy seems to also have an important role in the zygote and embryo and is likely involved in the elimination of paternal mitochondrial and other sperm derived organelles [19,20,21], although contradictory reports exist [22].

We also dedicated some studies to investigating autophagy in ejaculated mammalian spermatozoa, showing that autophagy, mitophagy, and autophagic-like molecular mechanisms are involved in regulating critical processes of sperm function, such as motility and viability [23], and they display a protective role in sperm during sperm cryopreservation and other biotechnological procedures [24,25].

In summary, autophagy components and processes have been described in spermatocytes, ejaculated spermatozoa, and the embryo after fertilization, but it has not been investigated so far whether autophagy-related processes are involved in the regulation of capacitation and the acrosome reaction in ejaculated mammalian spermatozoa. Therefore, in the present research work, we aimed: (1) to identify autophagy-related proteins in the acrosome of the stallion spermatozoa; (2) to study whether incubation in a capacitating media or the acrosome reaction can induce in these cells autophagy-like mechanisms, such as LC3 processing; and (3) to investigate the effect of autophagy inducers or inhibitors on sperm capacitation and acrosome reaction.

## 2. Results

### 2.1. LC3 Processing (LC3II/LC3I Ratio) during Sperm Capacitation

**Figure 1:**
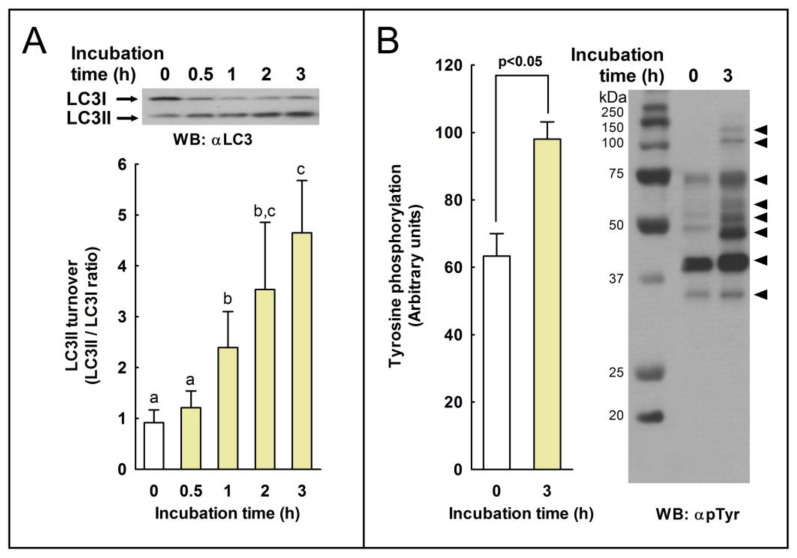
The effect of capacitation in LC3 turnover (LC3II/LC3I ratio) and protein tyrosine phosphorylation in stallion spermatozoa. (**A**) Equine spermatozoa were incubated in capacitating medium (BWW) for up to 3 h at 37 °C. At the indicated times, sperm cells were removed from the incubation vial and immediately lysed. Ten µg of proteins was loaded and resolved by SDS-PAGE, and immunoblotting was performed with an LC3 antibody (αLC3). The signal was quantified by densitometry, and LC3 turnover was expressed as the LC3II/LC3I ratio. Values represent the mean ± SEM of 6 independent experiments. Bars with different letters indicate significant differences (*p* < 0.05). (**B**) To assess the degree of capacitation, after 3 h of incubation in BWW at 37 °C, sperm cells were lysed in ice-cold lysis buffer supplemented with sodium orthovanadate, and protein tyrosine phosphorylation was investigated by using an anti-phosphotyrosine antibody (clone 4G10) (αpTyr). The signal was quantified by densitometry. Values on the left represent the mean ± SEM of 6 independent experiments (*p* < 0.05). On the right, a representative western blot is displayed. The bands show increased tyrosine-phosphorylation after incubation in capacitating medium (3 h) compared to control, and uncapacitated sperm cells (0 h) are indicated by the arrow heads in the right, showing, from top to bottom, 131, 113, 72, 62, 55, 48, 41, and 35 kDa.

To study whether the processing of LC3I to LC3II can occur in stallion spermatozoa during capacitation, samples were incubated at 37 °C in BWW for 3 h to induce sperm capacitation [26], and the degree of LC3 processing was estimated by western blotting with a commercial antibody that recognizes both the PE-conjugated form of LC3 (or LC3II; lower band) and the unconjugated form of LC3 (or LC3I; upper band) [25]. As shown in Figure 1, the incubation of sperm samples induced a significant increase in the LC3II/LC3I ratio after only 1 h of incubation, compared to time 0. This increment in the LC3II/LC3I ratio was a time-dependent process for up to 3 h of incubation. To confirm that stallion spermatozoa were experiencing capacitation, we measured protein tyrosine phosphorylation by western blotting in these samples, and we detected a marked increment in protein phosphorylation (Figure 1). This indicates that stallion spermatozoa were effectively experiencing capacitation after the incubation in BWW for 3 h, as previously described [26].

### 2.2. LC3 Processing during the Acrosome Reaction

**Figure 2:**
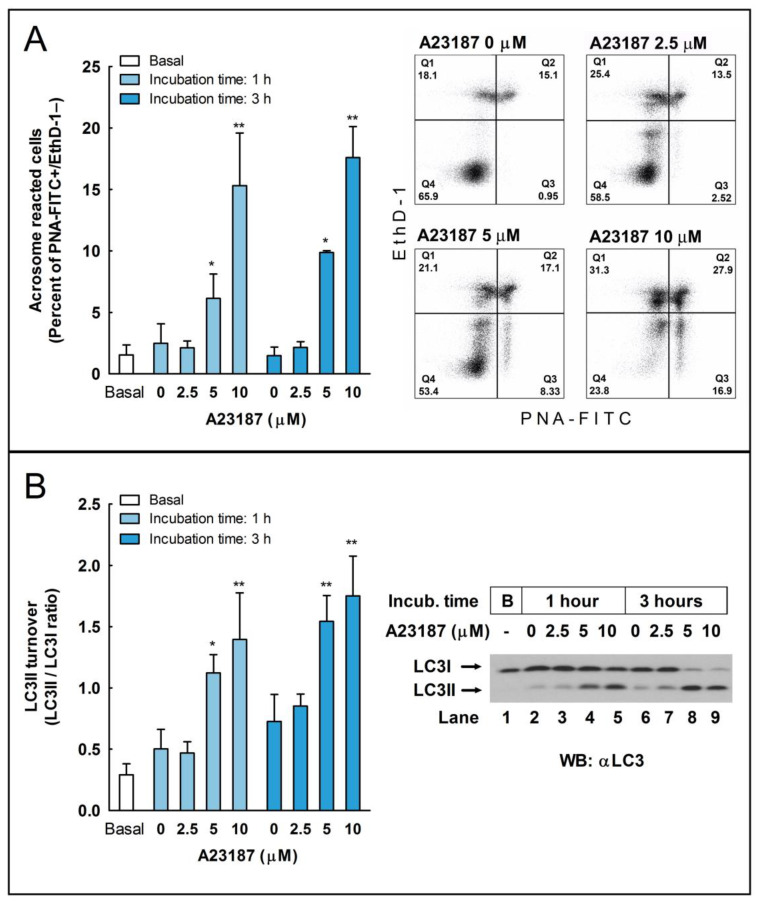
The induction of the acrosome reaction and enhanced LC3II processing by the calcium ionophore A23187 in stallion spermatozoa. (**A**) Ejaculated equine spermatozoa were immediately processed (basal) or preincubated for 30 or 150 min in BWW at 37 °C and then were challenged with the indicated concentrations of A23187 (2.5, 5, and 10 µM) or vehicle (0 µM; DMSO) for 30 additional min in the same incubation vial (the final incubation time in capacitating conditions were, respectively, 1 and 3 h). After incubation, spermatozoa were washed with PBS and labeled with probes to characterize by flow cytometry the DNA content (Hoechst 33342), sperm viability (Ethidium Homodimer-1; EthD-1), and acrosome status (Lectin from Arachis hypogaea FITC-conjugated; PNA-FITC). To select the sperm population, cytometer events were gated according to DNA content (Hoechst 33342+ events) and then were classified in four quadrants corresponding with the detection or not of each of the remaining fluorophores, resulting in dead unreacted cells (EthD-1+ and PNA-FITC−; Q1), dead reacted cells (EthD-1+ and PNA-FITC+; Q2), live reacted cells (EthD-1− and PNA-FITC+; Q3), and live unreacted cells (EthD-1− and PNA-FITC−; Q4). Results in the graph represent the mean ± SEM of six independent experiments expressed as a percentage of acrosome reacted live sperm cells (Q3 quadrant). Bars with an asterisk indicate significant differences compared to the control (vehicle) by a two-tailed Student’s t-test for unpaired samples (* *p* < 0.05; ** *p* < 0.01). In the right of the panel, representative density plots obtained are displayed of when stallion spermatozoa were capacitated for 150 min and then challenged during 30 further minutes with the indicated concentrations of A23187 or vehicle (0 µM A23187; DMSO) (total incubation time: 3 h). (**B**) Stallion spermatozoa were processed as above and basal (uncapacitated) or capacitated spermatozoa, with or without A23187, were washed with PBS and lysed in ice-cold lysis buffer. LC3I and LC3II were characterized by western blotting with an anti-LC3 antibody. The signal was quantified by densitometry, and LC3 turnover was expressed as the LC3II/LC3I ratio. Results in the graph represent the mean ± SEM of six independent experiments. Columns with an asterisk indicate significant differences compared to the control (vehicle, 0 µM A23187; DMSO) by a two-tailed Student’s t-test for unpaired samples (* *p* < 0.05; ** *p* < 0.01). In the right of the panel, a representative western blot is displayed, showing the LC3 status in the basal, uncapacitated, sperm population (B, Lane 1) or in sperm cells incubated at 37 °C for 1 h (Lanes 2–5) or 3 h (Lanes 6–9) and challenged during the last 30 min of incubation with either vehicle (0 µM A23187, Lanes 2 and 6) or the indicated concentrations of A23187 (2.5 µM, Lanes 3 and 7; 5 µM, Lanes 4 and 8; 10 µM, Lanes 5 and 9).

To investigate whether LC3 processing could be activated during the acrosome reaction in stallion spermatozoa, we incubated sperm cells with the calcium ionophore A23187 to induce the acrosome reaction [26], and we determined, by flow cytometry, the percent of acrosome-reacted sperm cells and, by western blotting, the LC3II/LC3I ratio (Figure 2). Results showed that, after the preincubation of sperm cells during 30 or 150 min in BWW at 37 °C, a further incubation for 30 min at 37 °C with A23187 (5 and 10 µM; lower concentrations were ineffective) caused a marked increase in the percent of viable cells undergoing the acrosome reaction, as detected by the labeling of these cells with the fluorescent probe PNA-FITC [27] (Figure 2A, compare Q3 quadrants). Concomitantly, with the evaluation of the acrosome reaction, a percent of cells from the same populations was lysed to evaluate the LC3II/LC3I ratio by western blotting. Results showed that the induction of the acrosome reaction also caused a remarkable and significant increase in the amount of LC3II (Figure 2B), indicating that these autophagic-like processes are activated during the acrosome reaction as during capacitation.

### 2.3. Identification of Autophagy-Related Proteins in Stallion Sperm and Subcellular Redistribution of LC3 during the Capacitation and the Acrosome Reaction

**Figure 3:**
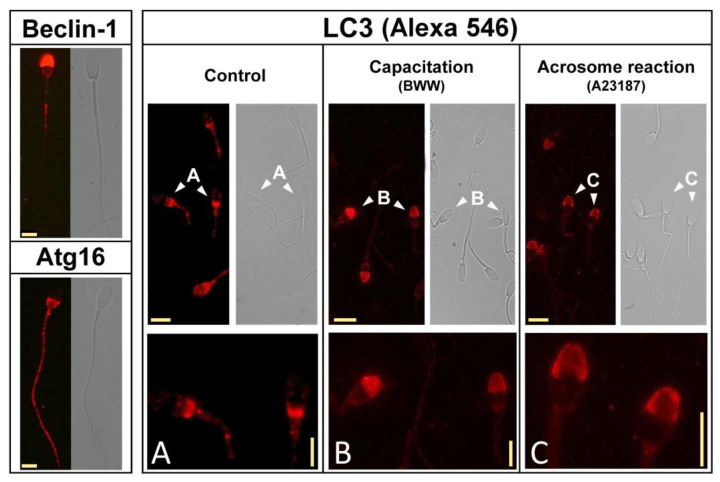
The immunolocalization of autophagy components in stallion spermatozoa and the subcellular redistribution of LC3 during capacitation and the acrosome reaction. The subcellular localization of autophagy proteins Beclin-1, Atg16, and LC3 was investigated by immunofluorescence in fixed and permeabilized stallion sperm using specific antibodies (see Materials and Methods section). LC3 subcellular distribution was studied in detail in stallion spermatozoa in basal conditions (Control, left panel), after incubation in BWW for 3 h at 37 °C (Capacitation, middle panel), and after incubation in BWW for 3 h at 37 °C with the addition of 5 µM A23187 during the last 30 min of incubation (Acrosome reaction, right panel). Bottom figures are representative areas digitally augmented to show the localization of LC3 in each respective panel corresponding to the cells indicated by the arrowheads ((**A**), control; (**B**), capacitation; (**C**), acrosome reaction), showing the three different patterns of LC3 distribution in sperm head. The horizontal yellow calibration bar represents 5 μm, and the vertical yellow calibration bar in the bottom represents 2.5 μm. The results are representative of 8 independent experiments.

Firstly, we aimed to study the subcellular localization of Beclin-1, Atg16, and LC3 by using immunofluorescence (see Materials and Methods section) with specific antibodies. Beclin-1 was immunodetected in unstimulated stallion sperm cells in the acrosomic region, with some labeling associated with the tail (Figure 3; top left panel), whereas Atg16 was primarily localized in the postacrosomic region and in the tail in these cells (Figure 3; bottom left panel). Next, we aimed to study the subcellular localization of LC3 with a specific anti-LC3 antibody, which recognizes both LC3I and LC3II. We investigated the distribution of LC3 in unstimulated stallion sperm cells, after the incubation in BWW for 3 h at 37 °C to induce capacitation, and after the induction of the acrosome reaction (Figure 3). In control cells, LC3 was primarily localized in the post-acrosomal region in the head and in the middle piece of the sperm tail (Figure 3; Control; Panel A). Upon incubation under capacitating conditions, LC3 immunolocalization redistributed into the head, showing an intense signal in the acrosome that virtually disappeared from the post-acrosomal region (Figure 3; Capacitation; Panel B). Finally, the induction of the acrosome reaction by the incubation of capacitated stallion spermatozoa (BWW; 150 min) with calcium ionophore A23187 (5 µM; 30 min) also caused an additional, although less evident, change in LC3 distribution, likely associated with acrosome membranes during the acrosome reaction (Figure 3; Acrosome reaction; Panel C).

### 2.4. Simultaneous Detection of LC3 and PNA in Control, Capacitated, and Acrosome-Reacted Stallion Sperm Cells by Confocal Microscopy

**Figure 4:**
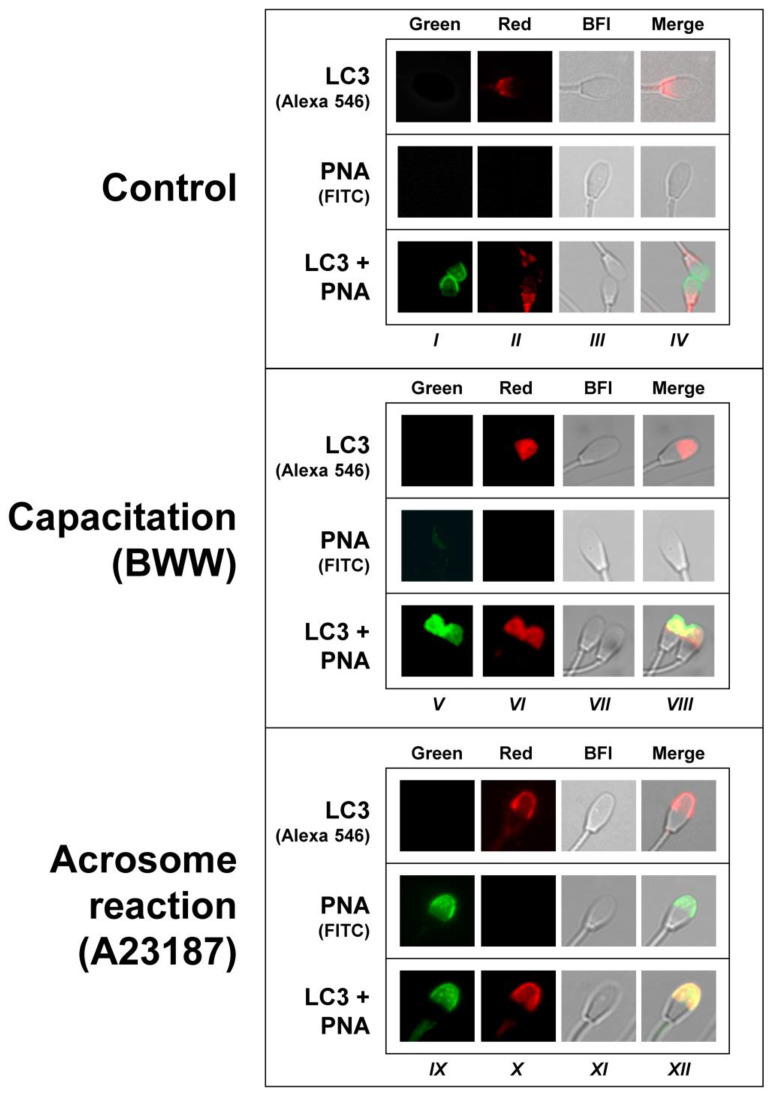
Simultaneous localization of LC3 and PNA in control, capacitated, and acrosome-reacted stallion sperm cells. Stallion spermatozoa were incubated in BWW for 3 h at 37 °C (Capacitation, Columns V to VIII), with the addition of 5 µM A23187 during the last 30 min of incubation (Acrosome reaction, Columns IX to XII), or they were processed without incubation (Control; Columns I to IV). The three populations (i.e., control, capacitated, and acrosome-reacted cells) were then incubated with a commercial anti-LC3 antibody followed by incubation with a secondary antibody labeled with Alexa 546 (LC3, Alexa 546), or they were labeled with an FITC-conjugated lectin from *Arachis hypogaea* (PNA-FITC) or with a combination of both probes (LC3 + PNA) (see Methods). The images were obtained with an inverted fluorescent microscope equipped with a 100× objective and the appropriate filters set to acquire green (Columns I, V, and IX) and red images (Columns II, VI, and X). Bright-field images (BFI, Columns III, VII, and XI) were also acquired. A superposition of these three images was constructed with the image processing software ImageJ (Merge, Columns IV, VIII, and XII), appearing yellow in the areas where the probes potentially colocalized. These images are the most representative (>80%) of the acrosome labeling in each population, although sperm cells showing different patterns in the acrosome (and outside the acrosome) were also occasionally observed. Differences in the green signal of the images obtained with the probe PNA-FICT alone or in combination with LC3 in the control and capacitated cells are due to the fact that the assay involving LC3 incubation required cell permeabilization, allowing PNA to label all the cells, not only those reacted, whereas in absence of the antibody (PNA alone), the cells were studied without permeabilization and PNA only labeled acrosome-reacted sperm cells, which were consistently below 2%. The experiment was repeated 4 times with 4 different stallions (N = 4).

Considering that LC3 likely associates with the acrosome membrane during the induction of the acrosome reaction in capacitated cells (Figure 3), treatment that significantly increased LC3I processing to LC3II (Figure 1 and Figure 2), we subsequently aimed to perform a more detailed study by using confocal microscopy to determine whether LC3 is associated or not with acrosome membranes during the acrosome reaction in stallion spermatozoa (Figure 4). Results showed that, as indicated in the previous figure, in control sperm cell LC3 immunolocalization was mainly associated with the post-acrosomal region in the head (Figure 4, Columns II and IV), whereas the PNA specific signal was undetectable in most of the cells (<2%). However, when PNA and LC3 labeling was performed concomitantly in permeabilized control cells (permeabilization is required to detect LC3), virtually all cells reacted to both PNA and LC3, with almost no overlapping of these signals within the sperm cells (Figure 4, bottom row in Control). This labeling patron clearly changed in capacitated (Figure 4, Columns V to VIII) and in the acrosome-reacted cells (Figure 4, Columns IX to XII). In agreement with the results obtained by means of classical fluorescence microscopy, when the capacitated sperms cells were challenged with 5 µM A23187 to induce the acrosome reaction, the LC3 specific signal in most cells (>80%) was immunodetected within the acrosome, likely associated with the membrane that clearly overlapped with the PNA signal detected in the permeabilized cells (Figure 4, bottom row in Acrosome reaction). The PNA labeling obtained in the permeabilized sperm was virtually identical to that obtained with PNA in the acrosome-reacted cells, although the signal was detected in a much lower cell proportion (≈10%) compared to permeabilized sperm cells (100%).

### 2.5. Colocalization Study of LC3 and PNA in Acrosome-Reacted Spermatozoa

**Figure 5:**
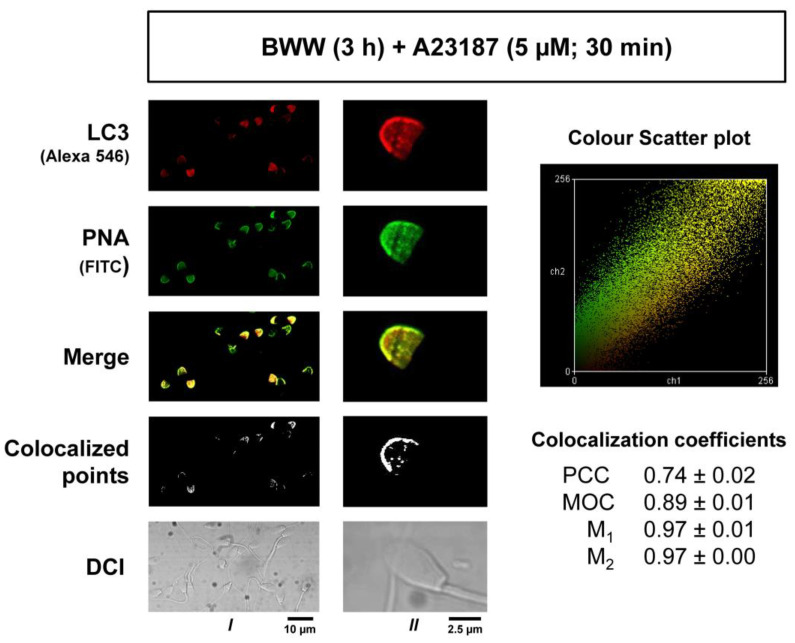
The colocalization of LC3 and PNA in acrosome-reacted stallion spermatozoa. Samples were processed as in Figure 4 to induce the acrosome reaction and then were permeabilized, labeled with an anti-LC3 antibody, and then simultaneously labeled with a fluorescent secondary antibody (Alexa 546) and FITC-labeled PNA, a lectin that specifically binds to the outer acrosome membrane. Red (LC3-Alexa 546) and green (PNA-FITC) fluorescence images were acquired with a Bio-Rad MRC-1024 confocal microscopy with a 60× objective and no digital zoom (Column I) to analyze the overall population or with a 100× objective and 1.2× digital augmentation of selected cells (Column II) to better analyze the probes within the acrosome. In both acquiring situations, a superposition of the red and green images was constructed with the image processing software ImageJ, appearing in yellow the areas where both probes colocalize (merge). A further colocalization analysis of these two probes was accomplished by using the colocalization plugin for ImageJ that highlights the colocalized points of red and green 8-bit images, appearing as white areas in the constructed image (colocalized points). Differential interference contrast images were also acquired (DIC) with the confocal microscopy to better illustrate the subcellular distribution of the probes. A second colocalization ImageJ plugin, namely JACoP, was used to generate a color scatter plot for each pair of red and green 8-bit images and a number of colocalization coefficients for these pairs, including Pearson’s correlation coefficient (PCC), Mander’s overlap coefficient (MOC), and Mander’s colocalization coefficients for channel 1 (M1) and channel 2 (M2) (see Methods). In the right part of the panel, the color scatter plot is displayed that was obtained after applying the JACoP plugin to the red and green images shown in column I. In the right part, there are also the PCC, MOC, M1, and M1 coefficients obtained after applying the JACoP plugin to 24 images obtained under the acquisition parameters used in column I. These coefficients are the mean ± SEM of 6 different sets of images from 4 independent experiments performed with different stallions (N = 4) and represent the colocalization analysis of LC3 and PNA in more than 400 sperm cells.

To determine the degree of association of LC3 with the acrosome membranes during the induction of capacitation, stallion sperm cells were processed to induce the acrosome reaction, and then LC3-derived red fluorescence and PNA-derived green fluorescence were analyzed to objectively quantify the signal overlapping by means of the software ImageJ assisted with two colocalization plugins (Figure 5). After the generation of a new overlapped image by the superposition of the red and green channels (Figure 5; Merge), the Colocalization plugin for ImageJ, which highlights as white pixels the areas where both signals were recorded, was first applied. Results showed that most of the LC3 red fluorescence overlapped with the PNA green signal and that both were located mainly in the membrane (Figure 5; Colocalized points). Second, a further colocalization analysis of green and red signals was constructed using the JACoP ImageJ plugin, which generated several colocalization coefficients based in the analysis of a color scatter plot for each pair of red and green images. These coefficients mathematically express the degree of colocalization and include the Pearson’s correlation coefficient (PCC), Mander’s overlap coefficient (MOC), and Mander’s colocalization coefficients for channel 1 (M1) and channel 2 (M2) [28]. After applying the analysis, PCC (0.74 ± 0.02), MOC (0.89 ± 0.01), M1 (0.97 ± 0.01), and M1 (0.97 ± 0.00) coefficients indicated that green and red fluorescence consistently overlapped in the images, indicating that LC3 is more than likely associated with the acrosome membrane during the acrosome reaction in stallion spermatozoa.

### 2.6. Effect of the Pharmacological Activation of Autophagy in the Regulation of the Acrosome Reaction

**Figure 6:**
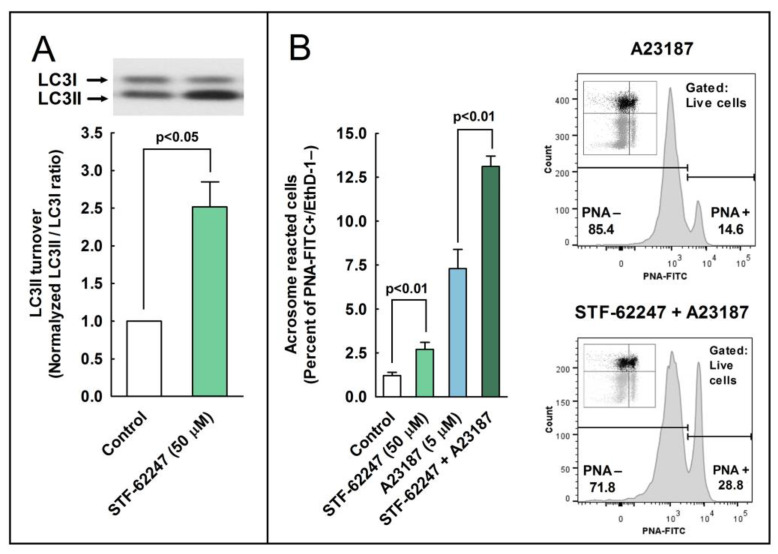
The effect of autophagy inducers on the processing of LC3II and the development of the acrosome reaction in stallion sperm cells. (**A**) Stallion spermatozoa were incubated for 3 h in BWW at 37 °C in the presence or absence (vehicle) of STF-62247 (50 µM). After incubation, LC3II processing from LC3I was assessed by western blotting and was expressed as normalized LC3II/LC3I ratio (control samples always equal 1). Values represent the mean ± SEM of 4 independent experiments. A two-tailed Student’s t-test for unpaired samples was applied to compare the means of each group with the significance level set at 0.05, as is indicated in the figure. In the top of the panel, there is an immunoblotting representative of three others. (**B**) Stallion spermatozoa were incubated for 150 min in BWW at 37 °C in the presence or absence (vehicle; DMSO) of STF-62247 (50 µM) and then were challenged with 5 µM A23187 or the vehicle (Control) for 30 additional min (the final incubation time in capacitating conditions was 180 min). After incubation, spermatozoa were washed with PBS and labeled with probes to characterize, by flow cytometry, the DNA content (Hoechst 33342), the cellular viability (EthD-1), and the acrosome reaction (PNA-FITC). To select the sperm population, cytometer events were selected by DNA content (Hoechst 33342+ events) and then were classified in four quadrants corresponding to the detection or not of each of the remaining fluorophores, resulting in dead unreacted cells (EthD-1+ and PNA-FITC−; Q1), dead reacted cells (EthD-1+ and PNA-FITC+; Q2), live reacted cells (EthD-1− and PNA-FITC+; Q3), and live unreacted cells (EthD-1− and PNA-FITC−; Q4). Results in the graph represent the mean ± SEM of 4 independent experiments expressed as the percentage of acrosome-reacted live sperm cells (Q3 quadrant). A two-tailed Student’s t-test for unpaired samples was applied to compare the mean of each treatment with their respective controls. The significance level was set at 0.01, as is indicated in the figure. In the right part of the panel, there is further analysis of the acrosome-reacted cells by gating out dead cells (EthD-1+). Therefore, only live cells (EthD-1−) were considered for this analysis. Representative histograms obtained from 5 µM A23187-treated cells in the presence (top) or absence (bottom) of 50 µM STF-62247 are shown. In these histograms, there are density plots of the same cytometric data displayed in quadrants, which were included as inserts. Only the gray areas from these inserts (Q3 and Q4, live cells) were used in their respective histograms, whereas the black areas were gated out for the analysis (Q1 and Q2, dead cells).

Our next objective was to investigate whether the activation of autophagy or autophagy-like processes by chemical inducers could also modulate the acrosome reaction in stallion spermatozoa. To address this question, we used STF-62247, a compound useful for the activation of autophagy at the final steps of the process [25]. Cells were incubated in BWW at 37 °C during 3 h in the presence of STF-62247 (50 µM), and control samples were incubated in BWW at 37 °C during 3 h with the vehicle (DMSO), as previously described [25]. LC3 processing was calculated as the ratio of LC3II/LC3I, and data were normalized with respect to the control (containing vehicle). In agreement with previous reports [25], results showed that the pharmacological activation of autophagy with STF-62247 significantly increased LC3 turnover (Figure 6A). To determine whether this compound could modulate the acrosome reaction, capacitated sperm cells were challenged with A23187 (5 µM) in the presence or absence of 50 µM STF 62247, and the percent of acrosome reacted cells was estimated by flow cytometry. Results showed that the incubation of sperm cells with STF-62247 significantly increased the percent of acrosome reacted cells (PNA+/EthD-), even in the absence of calcium ionophore (Figure 6B; compare control with STF-62247). This compound also increased the percentage of acrosome reacted cells by more than 60% after the incubation of spermatozoa with A23187 (Figure 6B, compare A23187 with STF-62247 + A23187) and by more than 100% when EthD-1+ cells were gated out and the acrosome reaction was only analyzed in live cells (Figure 6B, compare A23187 with STF-62247 + A23187 in the cytometer histograms).

### 2.7. Effect of the Pharmacological Inhibition of Autophagy in the Regulation of the Acrosome Reaction

**Figure 7:**
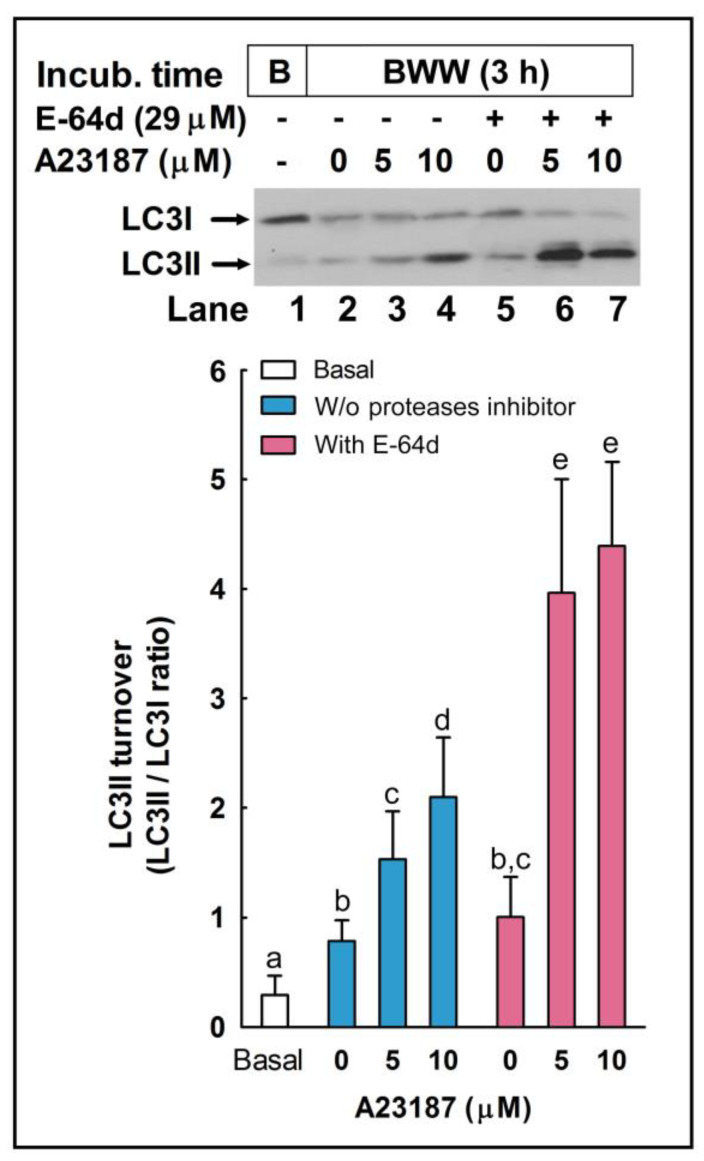
The effect of E64-d, a cathepsin inhibitor, on LC3II processing during the acrosome reaction in stallion spermatozoa. Equine spermatozoa were immediately processed (B; basal) or resuspended in BWW medium supplemented (lanes 2–4) or not (lanes 5–7) with the protease inhibitor E-64d (29 µM) for 150 min and then were challenged with the indicated concentrations of A23187 (5 and 10 µM) or the vehicle (0 µM; DMSO) for 30 additional min (the final incubation time in all samples, except basal, was 180 min). After the incubation, samples were lysed, and LC3 turnover was assessed by western blotting with an anti-LC3 antibody that recognizes LC3I and LC3II. Results were expressed as the LC3II/LC3I ratio. Values represent the mean ± SEM of 5 independent experiments. Bars with different letters show statistical differences by a two-tailed Student’s t-test for unpaired samples (*p* < 0.05).

**Figure 8:**
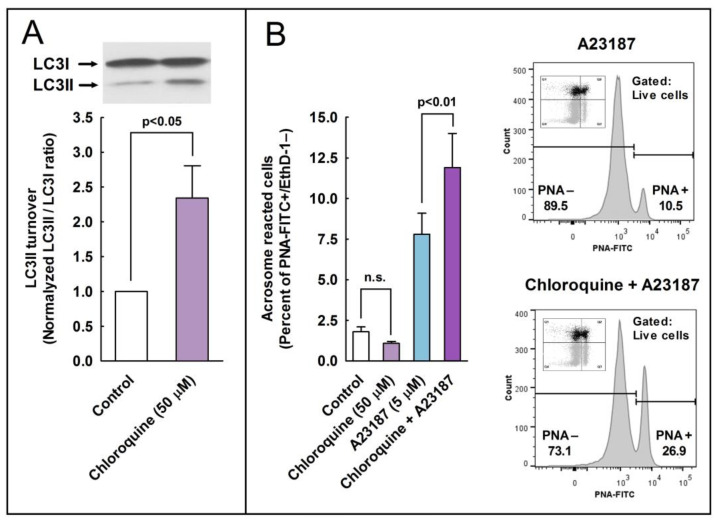
Effect of the autophagy inhibitor chloroquine on the processing of LC3II and the development of the acrosome reaction in stallion sperm cells. (**A**) Stallion spermatozoa were incubated for 3 h in BWW at 37 °C in the presence or absence of chloroquine (50 µM). After incubation, the processing of LC3I to LC3II was assessed by western blotting. LC3 turnover was expressed as the normalized LC3II/LC3I ratio (control samples always equal 1). Values represent the mean ± SEM of 4 independent experiments. A two-tailed Student’s t-test for unpaired samples was applied to compare the means of each group with the significance level set at 0.05, as is indicated in the figure. In the top of the panel, there is a representative immunoblotting of three other independent experiments. (**B**) Stallion spermatozoa were incubated for 150 min in BWW at 37 °C in the presence or absence (Control) of chloroquine (50 µM) and then were further incubated with 5 µM A23187 or the vehicle for 30 min (final incubation time 180 min). After incubation, spermatozoa were washed with PBS and labeled with probes to characterize, by flow cytometry, the DNA content (Hoechst 33342), cellular viability (EthD-1), and acrosome reaction (PNA-FITC). Cytometry data were processed as above, performing the analysis by quadrants (graph) or after gating out dead cells (histograms on the right). Results in the graph represent the mean ± SEM of 4 independent experiments expressed as the percentage of acrosome-reacted live sperm cells (Q3 quadrant). A two-tailed Student’s t-test for unpaired samples was applied to compare the mean of each treatment with their respective controls. The significance level is indicated in the figure.

**Figure 9:**
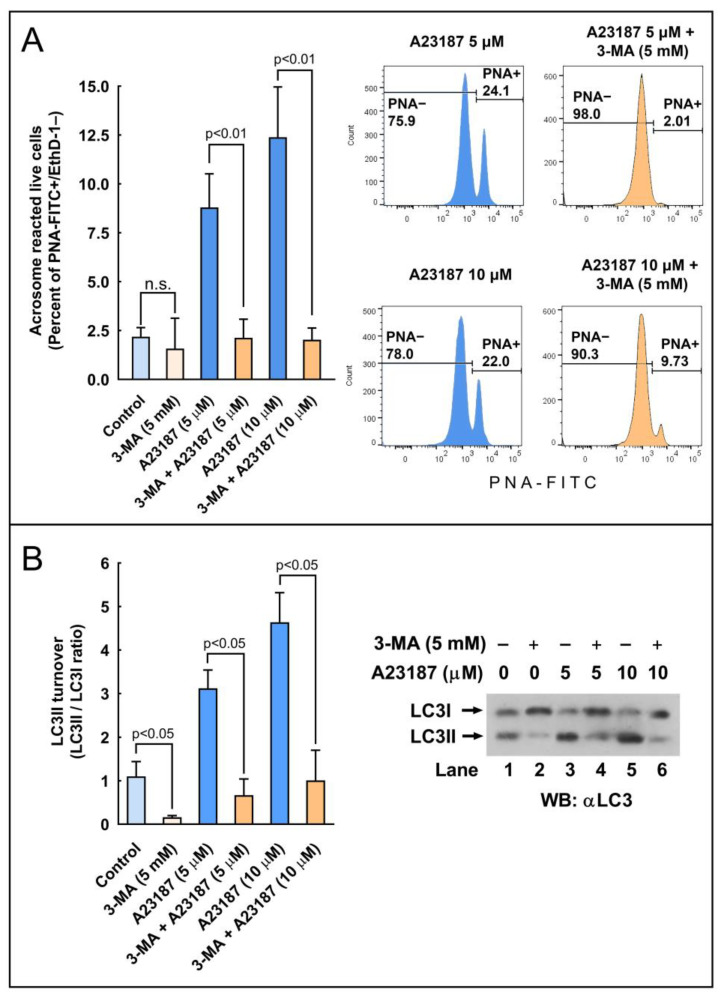
The effect of the autophagy inhibitor 3-methyladenine (3-MA) on the processing of LC3II and the development of the acrosome reaction in stallion sperm cells. (**A**) Stallion spermatozoa were incubated for 210 min in BWW at 37 °C in the presence or absence (control) of 3-MA (5 mM) and then were further incubated for 30 min (final incubation time 240 min) with the vehicle (control), 5 µM A23187, or 10 µM A23187. After incubation, spermatozoa were washed with PBS and labeled with probes to characterize, by flow cytometry, the DNA content (Hoechst 33342), cellular viability (EthD-1), and acrosome reaction (PNA-FITC). Cytometry data were processed as above, performing the analysis by quadrants (graph) or after gating out dead cells (histograms on the right). Results in the graph represent the mean ± SEM of 4 independent experiments expressed as the percentage of acrosome-reacted live sperm cells (Q3 quadrant). A two-tailed Student’s t-test for unpaired samples was applied to compare the mean of each treatment with their respective controls. The significance level is indicated in the figure. (**B**) Stallion spermatozoa were incubated for 4 h in BWW at 37 °C in the presence or absence of 3-MA (5 mM). After incubation, the processing of LC3I to LC3II was assessed by western blotting. LC3 turnover was expressed as the LC3II/LC3I ratio. Values represent the mean ± SEM of 4 independent experiments. A two-tailed Student’s t-test for unpaired samples was applied to compare the means of each group with the significance level set at 0.05, as is indicated in the figure. In the right part of the figure, there is a representative immunoblotting of three other independent experiments.

To further assess the role of autophagy-like processes in the regulation of the acrosome reaction in sperm cells, we used three pharmacological inhibitors of autophagy, namely E-64d, chloroquine, and 3-methyladenine (3-MA). E-64d is a membrane-permeable inhibitor of acid proteases, whereas chloroquine is a lysosomal lumen alkalizer. Both compounds function by suppressing lysosomal activity and, hence, are able to block the autophagic progress by impairing lysosomes and causing the accumulation of LC3II [29]. On the other hand, 3-MA is an inhibitor of class III phosphoinositide 3-kinase (PI3K), an ortholog of yeast Vps34, that plays a crucial role in an early step of autophagosome and in LC3 lipidation in mammalian cells [29]. The incubation of stallion spermatozoa in the presence of E-64d (10 μg/mL, 29 µM; 3 h) did not significantly increased the LC3II/LC3I ratio in the absence of calcium ionophore, but E 64d potentiated by more than 160% the LC3 turnover induced by 5 µM A23187 (Figure 7; compare lanes 3 and 6) and by more than 100% the LC3 turnover induced by 10 µM A23187 (Figure 7; compare lanes 4 and 7). Similarly, the incubation of sperm cells for 3 h at 37 °C in the presence of chloroquine (50 µM) was able to increase the LC3II/LC3I ratio by 1.32-fold in capacitated spermatozoa (Figure 8A). This treatment did not modify the percentage of acrosome-reacted cells in the absence of calcium ionophore (Figure 8B), but when chloroquine-treated cells were challenged with 5 µM A23187, the percentage of acrosome-reacted cells increased by around 60% compared to the response elicited by the calcium ionophore alone (Figure 8B; PNA+/EthD-) and by more than 150% when the acrosome reaction was analyzed only in live cells (Figure 8B, compare A23187 with chloroquine + A23187 in the cytometer histograms). Interestingly, the incubation of stallion spermatozoa in the presence of 3-MA (5 mM; 4 h) almost completely inhibited the acrosome exocytosis triggered by 5 µM or 10 µM A23187 (Figure 9A). Such treatment also caused the notable inhibition of the LC3II/LC3I ratio induced by A231987 (Figure 9B) and also significantly inhibited the LC3 ratio in capacitated spermatozoa (Figure 9B; compare lanes 1 and 2).

## 3. Discussion

In previous reports, we demonstrated that human and stallion ejaculated spermatozoa express a battery of proteins related to autophagy initiation and progression, such as Atg5, Atg16, Beclin-1, and LC3 [23,24,25,30]. In these reports, we showed that autophagy, mitophagy, and autophagic-like molecular mechanisms are activated ex vivo and are involved in the regulation of critical processes of sperm function, such as motility and viability [23], and they exert a protective role in sperm during cryopreservation and other biotechnological procedures [24,25,30]. These seminal reports were confirmed and extended by posterior studies in other laboratories [31,32,33,34]. Therefore, today, it seems clear that autophagy is not only essential for sperm development during spermatogenesis, but it also might play critical roles ex vivo in adult, ejaculated spermatozoa once the spermatogenesis and the epidydimal maturation has been completed (reviewed in [10]).

In this report, we investigated additional functions and cellular processes that could be regulated by the molecular machinery associated with autophagy. Specifically, we investigated the putative participation of the autophagy component LC3 in the acrosome reaction in stallion spermatozoa. LC3 is the autophagosomal ortholog of yeast Atg8 and is present in somatic cells in two forms, LC3I and LC3II. The unprocessed form LC3I is localized in cytosol and, after the canonical autophagy activation, is conjugated to phosphatidylethanolamine to form LC3-phosphatidylethanolamine conjugate (Atg-8-PE, LC3-PE or LC3II), which is recruited to autophagosomal membranes [35]. LC3II is considered an autophagosomal marker in mammals, and it is widely used for monitoring canonical autophagy flux in mammalian somatic cells [1,2,6,7], as well as in mammalian spermatozoa [23,25,32]. LC3 is also crucial in regulating pathways leading to non-canonical functions of autophagy machinery, including LC3-associated phagocytosis and LC3-associated endocytosis [8].

We studied whether autophagy-related processes are active or not in these cells during sperm capacitation by studying by western blotting the conversion of LC3I to LC3II (LC3II/LC3I ratio). In somatic cells, autophagy is induced mainly by the deprivation of nutrients over time by oxidative stress, by virus infection and replication, or by many other stressful situations in the cell as a survival mechanism and in response to a number of cellular stresses [2,11,36]. In stallion spermatozoa, we observed a significant increase in LC3 processing only after 1 h of incubation in capacitating conditions (Figure 1), indicating that LC3 processing might be achieved in the sperm cell ex vivo upon normal physiological situations. Capacitation is a prerequisite of the acrosome reaction [26,37]. Our next objective was to investigate whether the activation of the pathways leading to LC3 processing during capacitation could also be activated during the acrosome reaction or, in opposition, were independent mechanisms. To address this question, we challenged capacitated stallion spermatozoa with the calcium ionophore A23187, a well-known inductor of the acrosome reaction in these cells [26]. We found that this treatment increased both the percentage of acrosome-reacted cells, as expected, but also caused a striking increment in the LC3II/LCI ratio compared to the untreated cells (Figure 2).

Next, we aimed to investigate whether the pharmacological activation or inhibition of the autophagy pathways could also stimulate or avoid the acrosome reaction in the spermatozoa. To address these questions, we employed compounds widely used in somatic cells as canonical autophagy activators (STF 62247) [29,38] or inhibitors (E-64d, chloroquine, and 3-MA) [29,39]. In stallion spermatozoa, STF-62247 induced a significant increment in LC3I processing to LC3II and in the precent of acrosome-reacted cells. On the other hand, the inhibition of autophagy with E 64d also significantly increased the processing of LC3I to LC3II, whereas chloroquine increased LC3 turnover as well and also the percentage of acrosome-reacted cells. In somatic cells, chloroquine inhibited autophagy as it raised the lysosomal pH, whereas STF-62247 stimulated autophagy because it acidified the pH in the same organelle [29]. These results, with STF-62247 and chloroquine increasing the ratio of LC3II/LC3I and the acrosome reaction, are not easily interpretable since both inhibitors display opposing mechanisms of action. However, the increment in the LC3II/LC3I ratio is possible because it accelerated the transition from LC3I to LC3II, or because LC3II is not degraded by proteolysis and accumulates [7]. One way or another, it seems that variations in the pH of the acrosomal lumen are not enough to block or slow down LC3 processing induced by calcium ionophore or to block or reduce the percentage of cells experiencing the acrosome reaction. It seems also clear that, since both processes take place simultaneously, the processing of LC3II might be an important step in the development of the acrosome reaction. This was further clarified by the results with 3-MA, a compound that was able to inhibit the LC3 turnover concomitantly with the almost complete inhibition of the acrosome exocytosis induced by calcium ionophore. All these results likely indicate that a relationship between LC3 and the acrosome reaction exists and that this turnover (LC3 lipidation) is probably essential to trigger the acrosome exocytosis in mammalian spermatozoa.

As has been described in somatic cells, the inhibition of the proteolytic degradation of LC3II seems to be responsible for the increased LC3 ratio in the samples treated with E-64d, a membrane-permeable inhibitor of acid proteases [29], very abundant in lysosomes in somatic cells and in acrosome in sperm cells [40]. The accumulation of LC3II upon the inhibition of acrosomal proteases could indicate that LC3II is proteolytically degraded in the acrosome during the acrosome reaction, similarly to the mechanism described in the autophagosome in somatic cells, where LC3 is cleaved by lysosomal proteases during canonical autophagy [1,2].

After we defined the ability of capacitation and calcium ionophore to accelerate the processing of LC3, we investigated the subcellular distribution of LC3 and its transitions during such processes in the spermatozoa. We found that this protein has at least three different patterns of distribution along the sperm head, which change during sperm activation and might suggest that LC3 translocates to be recruited to target membranes [35]. In mammalian spermatozoa, the translocation of proteins seems completely necessary for its interaction with other proteins and, therefore, for triggering a proper signaling cascade [41,42]. In fresh uncapacitated spermatozoa, where the LC3II/LC3I ratio is low, most of the sperm cells were stained in the postacrosomic region. After the incubation of spermatozoa in capacitating conditions, we detected a redistribution of LC3 proteins from the postacrosomic to the acrosomic region, indicating that LC3 is actively processed in normal ejaculated spermatozoa after a physiological challenge and that LC3 processing is not a mere reminiscence from spermatogenesis. In the next step, we saw that LC3 colocalized with PNA-FITC (Figure 4 and Figure 5). PNA-FITC and PSA-FITC are the most routinely used lectins to label the acrosome. PNA lectin was shown to bind Galß(1–3)Gal NAC residues located on the outer acrosomal membrane, whilst PSA was reported to recognize α-methyl mannoside residues from complex oligosaccharide structures, localized within the acrosome contents [43]. At working concentrations, they do not bind to intact acrosomes unless cells have been previously permeabilized, giving a specific comparison between unreacted and reacted sperm [44]. Results in our experiments, where PNA-FITC and LC3 signals clearly colocalized, indicate that, when LC3 is associated with PE, it can be recruited to the acrosomal membrane or to the apical plasmalemma membrane that is surrounding the acrosome. In somatic cells, LC3 lipidation (PE conjugation, or the transition of LC3I to LC3II) is a critical and necessary step to associate to the autophagosome membranes, and only LC3II is associated with the membranes during canonical [1,2,35] as well as non-canonical autophagy [8].

The immunoreactivity of additional components related to autophagy that were investigated in our study was mainly detected in the tail and in the head, associated or not to the acrosome (Figure 3). In somatic cells, it was described that LC3 processing (i.e., the conversion from LC3I to LC3II) requires several Atg and related proteins, which form multimolecular complexes involved in the processing of LC3I to LC3II, which ultimately associates with biological membranes (typically with autophagosomes) [35,45]. In our study, Beclin-1 colocalized with LC3 in the acrosome, whereas Atg16 was mainly located in the tail and postacrosome in the head. In mature cells, LC3 was located in the middle piece but primarily can be found in the head. In most of the cells in the untreated population, LC3 was located in the postacrosomal region, where it colocalized with Atg16. Upon capacitation, LC3 was mainly detected in the acrosome, where it colocalized with Beclin-1. It is worth mentioning that, upon its association with PI3K, Beclin-1 is also associated with the membrane [45,46], and the final step in LC3 processing, namely PA conjugation and membrane association, is mediated, among other proteins, by Atg7, which is also related to acrosome membranes and LC3 processing in murine precursor sperm cells [16]. Therefore, when considering that Beclin-1/PI3K complexes perform their functions in somatic cells after the Atg16 nucleated complexes, it can be easily speculated that LC3 translocation from the postacrosome region to the acrosome upon sperm activation, where more than likely LC3 is processed to LC3II and is associated with the acrosome or sperm apical membranes, is mediated by the sequential activation of Atg16- and Beclin-1-bearing complexes. Such a hypothesis involves an earlier step requiring Atg16 (postacrosome) and a subsequent step involving Beclin-1/PI3K (acrosome) and, based on the literature [2,16], possibly also Atg7. To completely elucidate whether those mechanisms effectively take place during LC3 processing in stallion spermatozoa obviously requires additional studies, but so far, the localization of proteins, both in basal as well in activated sperm cells, is fully compatible with the described mechanisms in somatic cells.

In conclusion, we described additional processes that could be regulated by the autophagy components in sperm cells ex vivo. The LC3 ratio rapidly increases during sperm capacitation and, more intensely, during the acrosome reaction. During capacitation, LC3 translocate in the sperm head from the proacrosomal region to the acrosome, and later, during the acrosome reaction, LC3 seems to colocalize with the apical plasmalemma or with the acrosome membrane. Given these results, it can be speculated that, in stallion sperm, a form of noncanonical autophagy utilizes some components of autophagy machinery to facilitate the acrosome reaction. In support of this hypothesis, there is the fact that the acrosome is an organelle that shows a high resemblance to lysosomes [40], and accumulating evidence implicates LC3 and other autophagy-related proteins in cellular secretion [47,48]. Therefore, it can be speculated that autophagy proteins and related pathways could play a role in the fusion of the acrosome, a lysosome-derived vacuole, to the plasmalemma during the acrosome reaction, participating in a process that has been described as a secretory lysosome [40,49]. Considering that, in the spermatozoa, the process of canonical autophagy (the formation of an autophagolysosome) is only possible in a sizeable environment of the cytoplasmic droplet and given the origin and functional organization of the acrosome [50], the implication of LC3 in acrosome exocytosis is perhaps one of the functions more easily attributable to these autophagy components in mammalian sperm cells. However, the participation or not of a non-canonical form of autophagy in the regulation of these processes deserves further studies.

## 4. Materials and Methods

### 4.1. Reagents

Chemical salts (NaCl, KCl, MgSO4, KPO4, sodium pyruvate, sodium lactate, CaCl_2_, NaHCO_3_, Na_3_VO_4_), HEPES, EGTA, EDTA, deoxycholate, Triton X-100, glucose, bovine serum albumin (BSA), formaldehyde, phosphate buffered saline (PBS), chloroquine diphosphate salt (Ref# C6628), sterile-filtered dimethyl sulfoxide (DMSO; Ref# D2650), E-64d (Aloxistatin) (Ref# E8640), and anti-LC3B polyclonal antibody (Ref# L7543) were purchased from Sigma-Aldrich (Madrid, Spain). 3-Methyladenine (Ref# S2767) was purchased from Selleck Chemicals (Planegg, Germany). Anti-Beclin-1 pAb (Ref# PD017Y), Anti-Atg16 pAb (Ref# PM040Y), and a positive control for anti-LC3 (Ref# PM036-PNY) were purchased from MBL International (Woburn, MA, USA). EthD-1 and the anti-rabbit IgG antibody labeled with Alexa 546 were obtained from Molecular Probes (Thermo Fisher Scientific, Madrid, Spain). Anti-rabbit IgG horseradish peroxidase (HRP)-conjugated secondary antibody and enhanced chemiluminescence detection reagents were from Pierce Protein Biology (Thermo Fisher Scientific, Madrid, Spain). A complete, EDTA-free, protease inhibitor cocktail was from Roche Diagnostics (Barcelona, Spain). Bradford reagent, Tris/Glycine/SDS buffer (10×), and Tris/Glycine buffer (10×) were from Bio-Rad (Madrid, Spain). STF-62247 (Ref# 189497) was from Merck Millipore (Darmstadt, Germany).

### 4.2. Collection of Equine Semen

Semen was obtained from a total of 14 Andalusian horses aged between 4 and 12 years that were housed in individual stables at the Veterinary Teaching Hospital of the University of Extremadura, Caceres, Spain. Stallions were maintained according to institutional and European regulations. All animals were healthy sperm donors of proven fertility and were collected on a regular basis (three collections per week) during the breeding season for AI. Ejaculates were collected using a Missouri model artificial vagina with an inline filter to eliminate the gel fraction, and it was lubricated and warmed to 45–50 °C. Semen was immediately evaluated and processed.

### 4.3. Incubation Media and Treatments

To perform the incubation of semen samples, for each stallion’s ejaculate, aliquots were centrifuged at 800× *g* for 10 min at RT. Seminal plasma was removed and the sperm pellet resuspended to 100 × 10^6^ spermatozoa mL-1 in modified Biggers, Whitten, and Whittingham (BWW) media consisting of: 91.6 mM NaCl, 4.6 mM KCl, 2.44 mM MgSO_4_, 1.2 mM KPO_4_, 20 mM HEPES, 5.6 mM glucose (anhydrous), 0.27 mM sodium pyruvate, 44 mM sodium lactate, 1.7 mM CaCl_2_, 25 mM, NaHCO_3_, and 7 mg/mL BSA. The pH of the solution was adjusted to 7.4, and osmotic concentration was kept at 320 mOsm/kg.

### 4.4. Autophagy Regulation

Samples were incubated 3 h at 37 °C in the presence of autophagy modulators, namely STF-62247 (50 µM), chloroquine (50 µM), and E-64d (10 µg/mL; 29 µM) or for 4 h at 37 °C with 3-MA (5 mM). STF-62247 was described as a selective autophagy activator, whereas chloroquine, 3-MA, and E-64d inhibit autophagy [29]. Chloroquine raises the lysosomal pH, which leads to the inhibition of both fusion of autophagosome with lysosomes and lysosomal protein degradation; 3-MA inhibits class III Pi3K, impairing the signaling leading to LC3 processing; and E-64d is a membrane-permeable and irreversible inhibitor of cathepsins [29]. The vehicle for STF and E-64d was DMSO, which was systematically added to the controlled sperm population at the maximal concentration used (0.004%; *v*/*v*). Chloroquine is water-soluble; therefore, the control population was incubated in the absence of vehicle; 3-MA is also water soluble at the working concentration and was dissolved directly in the incubating medium (BWW), prewarmed at 45 °C to facilitate its solubilization. The 3-MA-containing medium was cooled down to 37 °C before use.

### 4.5. Western Blotting

To separate the proteins according to their apparent molecular masses, SDS-PAGE was performed as previously described [51] with minor modifications. Samples (1 mL) were washed with Phosphate-buffered saline (PBS), resuspended in lysis buffer (50 mM Trizma base, 150 mM NaCl, 1% Triton X-100, 1% deoxycholate, 1 mM EGTA, 0.4 mM EDTA), supplemented with a protease inhibitor cocktail and phosphatase inhibitor (0.2 mM Na3VO4), and sonicated for 5 s at 4 °C. The homogenate was centrifuged for 15 min at 10,000× *g* at 4 °C, and the protein concentration was estimated in the supernatant, containing soluble proteins in non-ionic and ionic detergents. Proteins were mixed with loading buffer supplemented with 5% mercaptoethanol and further denatured by heating for 10 min at 70 °C. Ten to fifteen micrograms of protein extract from mammalian spermatozoa or positive controls were loaded and resolved by SDS PAGE on a 12% polyacrylamide gel. The proteins were then transferred to a nitrocellulose membrane, which was subsequently blocked with blocking buffer (5% non-fat dry milk) in a Tris buffered saline with Tween-20 (TBST) (10 mM Trizma base, 100 mM NaCl, and 0.05% Tween 20) for 1 h at RT. Immunoblotting was performed by incubating the membranes in blocking buffer overnight at 4 °C with the primary antibodies against LC3 and tyrosine-phosphorylated proteins. Membranes were then washed in TBST and incubated with goat anti-rabbit horseradish peroxidase-conjugated secondary antibody for 45 min at RT. Following 3 washes with TBST for 10 min each, the signal was visualized using a SuperSignal West Pico Chemiluminescent Substrate Kit according to the manufacturer’s instructions. Band intensity was quantified using the software Scion Image for Windows, version 4.02 (Scion Corp., Frederick, MD, USA).

### 4.6. Assessment of Acrosome-Reacted Spermatozoa and Cell Viability

One mL of sperm suspension (5 × 10^6^ spermatozoa mL^−1^) was loaded with Hoechst 33342 (0.5 μM). After thorough mixing, the sperm suspension was incubated at RT in the dark for 25 min. The suspension was then washed and resuspended with PBS and stained with PNA-FITC (25 µM). After thorough mixing, the sperm suspension (1 mL) was incubated at 37 °C in the dark for 15 min. Finally, ethidium homodimer-1 (EthD-1) (1.167 mM) was added to the sperm suspension during the last 5 min of incubation. During the analysis, this staining allowed us to exclude debris and non-sperm events (Hoechst, see below) and distinguished four sperm subpopulations [27]. The subpopulation of unstained spermatozoa was considered alive with intact acrosomes (unreacted cells). Biological membranes in these cells are impermeable to PNA and EthD-1; therefore, these cells remained unlabeled. The acrosome membrane was only available in acrosome-reacted cells, whose plasmalemma fused with the external acrosome membrane, allowing the PNA to enter to the acrosome. Consequently, the PNA-FITC positive subpopulation, emitting green fluorescence, corresponded to the acrosome-reacted spermatozoa. Finally, two additional subpopulations were easily detected, corresponding to spermatozoa stained with both EthD-1 and PNA-FITC (emitting green and red fluorescence) and spermatozoa stained with EthD-1 (emitting only red fluorescence). Both subpopulations were dead, unviable spermatozoa. Flow cytometric analyses were performed using an MACSQuant Analyser 10 (Miltenyi Biotech) flow cytometer, equipped with 3 lasers emitting at 405, 488, and 635 nm. The system was controlled using MACSQuantify Software. Sperm subpopulations were divided by quadrants to quantify the frequency of each subpopulation. Forward and sideways light scatter were recorded for a total of 30,000 events per sample. Non-sperm events were eliminated by gating the sperm population after Hoechst 33342 staining. All positive events for Hoechst 33342 within a certain range of the side scatter (SCC) distribution were considered spermatozoa. The compensation of spectral overlap was performed before each experiment using negative (unstained) and positive (single stained) controls for each single-stained compensation control sample. Additionally, thresholds for each quadrant were determined using unstained, isotype controls and single-stained control samples for each of the probes used in each individual experiment.

### 4.7. Indirect Immunofluorescence with Classical Microscopy and with Confocal Microscopy

After the incubation under the required conditions, spermatozoa were washed twice with PBS and then processed for immunofluorescence. Fifteen µL of pretreated semen samples were spread on poly-L-lysine-coated glass-bottom chambers (Nunc Ref#155411) and allowed to attach for 10 min. Spermatozoa were fixed with 4% formaldehyde in PBS for 15 min at RT and permeabilized with 0.2% Triton X-100 (*v*/*v*) in PBS for 5 min. Cells were washed three times for 10 min with PBS and incubated in PBS supplemented with 5% BSA (*w*/*v*) for 90 min to block nonspecific sites. After blocking, slides were incubated with primary antibodies (LC3) overnight at 4 °C, diluted in PBS containing 5% BSA (*w*/*v*). The following day, samples were extensively washed with PBS and further incubated for 45 min at RT with the secondary antibody diluted to 1/500 in PBS containing 5% BSA (*w*/*v*), consisting of an anti-rabbit antibody conjugated with the Alexa 546 probe. In the colocalization studies, the Alexa 546-labeled anti-rabbit secondary antibody was used simultaneously with PNA-FITC, for which the incubation conditions for the latter were identical to those used in flow cytometry (25 µM; 15 min at RT). Finally, the slides were thoroughly washed with PBS and examined. The images were obtained with a Nikon Eclipse TE200 inverted fluorescent microscope equipped with a 100× objective in oil immersion, and the appropriate filter was set to acquire green and red images. Bright-field images (BFI) were also acquired. In the colocalization experiments, the slides were also examined in a Bio-Rad MRC1024 confocal microscope with 60× and 100× objectives, both in oil immersion. The absence of nonspecific immunolabeling was assessed by processing the samples without primary antibodies.

### 4.8. Colocalization Study

The images were acquired with a Bio-Rad MRC1024 confocal microscope, as described above, with the appropriate filter set to obtain green and red channels for each acquisition, corresponding, respectively, with PNA-FITC and LC3-Alexa 546. Differential interference contrast images (DCI) were also obtained. A superposition of the red and green images was initially constructed with the image processing software ImageJ [52], by using the *merge channels* function. The images were further analyzed with the ImageJ software with the addition of two plugins, namely “Colocalization” and “JACoP”, which were obtained in the plugin repository page (https://imagej.nih.gov/ij/plugins) (last access on 20 December 2022). The first one was used to generate a qualitative visualization analysis, which highlighted the colocalized points of red and green 8-bit images, appearing as white areas in the constructed image. The second plugin is a compilation of co-localization tools, which calculated a set of commonly used co-localization indicators [28]. In this study, we used Pearson’s correlation coefficient (PCC), Mander’s overlap coefficient (MOC), and Mander’s colocalization coefficients for channel 1 (M1) and channel 2 (M2) for each obtained green/red image pair, without modifying the automatic threshold in any channel or applying ROIs. The values for PCC ranged from +1 to −1. A value of +1 represents perfect correlation, whereas −1 theoretically represents perfect exclusion, and 0 (zero) represents random localization in a situation where the labeling of both fluorochromes was proportional to the other and the detection of both was assessed in a linear range [28]. Low (close to zero) and especially negative PCC values for fluorescent images can be difficult to interpret in terms of colocalization. However, a value close to 1 does indicate reliable colocalization and constitutes the first good quantitative estimation of this process [28]. MOC is based on PCC, with average intensity values being taken out of the mathematical expression [28]. This coefficient varies from 0 to 1, the first corresponding to non-overlapping images and the latter reflecting 100% colocalization between both images. Finally, M1 and M2 are the split coefficients of the Mander’s colocalization coefficients for channel 1 and 2, respectively, indicating the fraction of green channel overlapping red (M1) and the fraction of red channel overlapping green (M2). These split coefficients avoid issues relating to absolute intensities of the signal, since they are normalized against total pixel intensity, and also provide information about how well each channel overlaps the other [28]. The obtained data for each coefficient, namely PCC, MOC, M1, and M2, were tabled accordingly with their acquisition conditions and treatments, and the mean ± SEM was calculated and used as a quantitative estimation of the LC3/PNA colocalization map after the induction of the acrosome reaction.

### 4.9. Statistical Analysis

Data were first examined using the Kolmogorov–Smirnov test to determine their distribution. Multivariate analysis of variance was performed (ANOVA) followed by post hoc Tukey’s test. If data did not adjust to a normal distribution, the nonparametric Mann–Whitney U-test was used to compare pairs of values directly. All analyses were performed using SPSS version 17.0 for Windows (SPSS Inc., Chicago, IL, USA). Statistical significance was set at *p* < 0.05.

## Data Availability

Not applicable.
